# Mutational Analysis of RIP Type I Dianthin-30 Suggests a Role for Arg24 in Endocytosis

**DOI:** 10.3390/toxins16050219

**Published:** 2024-05-10

**Authors:** Louisa Schlaak, Christoph Weise, Benno Kuropka, Alexander Weng

**Affiliations:** 1 Institute of Pharmacy, Freie Universität Berlin, Königin-Luise-Str. 2+4, 14195 Berlin, Germany; l.schlaak@fu-berlin.de; 2 Institute of Chemistry and Biochemistry, Freie Universität Berlin, Thielallee 63, 14195 Berlin, Germany; chris.weise@biochemie.fu-berlin.de (C.W.); kuropka@zedat.fu-berlin.de (B.K.)

**Keywords:** ribosome-inactivating proteins, type I RIP, *N*-glycosylase, triterpene saponins, endosomal escape enhancer, basic amino acid residues, endocytosis

## Abstract

Saponin-mediated endosomal escape is a mechanism that increases the cytotoxicity of type I ribosome-inactivating proteins (type I RIPs). In order to actualize their cytotoxicity, type I RIPs must be released into the cytosol after endocytosis. Without release from the endosomes, type I RIPs are largely degraded and cannot exert their cytotoxic effects. Certain triterpene saponins are able to induce the endosomal escape of these type I RIPs, thus increasing their cytotoxicity. However, the molecular mechanism underlying the endosomal escape enhancement of type I RIPs by triterpene saponins has not been fully elucidated. In this report, we investigate the involvement of the basic amino acid residues of dianthin-30, a type I RIP isolated from the plant *Dianthus caryophyllus* L., in endosomal escape enhancement using alanine scanning. Therefore, we designed 19 alanine mutants of dianthin-30. Each mutant was combined with SO1861, a triterpene saponin isolated from the roots of *Saponaria officinalis* L., and subjected to a cytotoxicity screening in Neuro-2A cells. Cytotoxic screening revealed that dianthin-30 mutants with lysine substitutions did not impair the endosomal escape enhancement. There was one particular mutant dianthin, Arg24Ala, that exhibited significantly reduced synergistic cytotoxicity in three mammalian cell lines. However, this reduction was not based on an altered interaction with SO1861. It was, rather, due to the impaired endocytosis of dianthin Arg24Ala into the cells.

## 1. Introduction

Ribosome-inactivating proteins (RIPs) are plant toxins that exhibit characteristic *N*-glycosylase activity (EC 3.2.2.22). RIPs inhibit protein synthesis by releasing a particular adenine residue from 28S ribosomal RNA (at position 4324, related to rats) and thus provoke cell death [1,2]. They are divided into two main types. Type II RIPs are polypeptide proteins with two domains, consisting of an enzymatic A domain that exerts *N*-glycosylase activity and a B domain. The B domain exhibits lectin-like properties that mediate binding to terminal *N*-acetylgalactosamine or galactose residues on the cell surface [3]. Therefore, they regulate uptake into the cell. The best-known type II RIP is ricin, a dimeric protein isolated from the seeds of *Ricinus communis* L. (Euphorbiaceae), whose uptake and transport in the cell has been studied extensively: the ricin B domain, with its lectin-like properties, enables binding to the cell surface and subsequent receptor-mediated endocytosis into the cell [3]. Consequently, dimeric ricin is transported to the endoplasmic reticulum (ER) by retrograde transport via the Golgi apparatus. At the ER, the two domains dissociate by reducing the disulfide bond [4]. The ricin A domain is then translocated to the cytosol, where it is able to exert its characteristic *N*-glycosylase activity [5,6,7]. 

Type I RIPs consist of a single peptide chain (A domain) that exerts enzymatic activity in the same manner as type II RIPs. They are differentiated by the absence of a cell-binding B domain [8]. Consequently, type I and type II RIPs are transported through the cell via differing routes [9]. The uptake mechanism of type I RIPs has so far been less extensively characterized [10]. Different endocytosis mechanisms for type I RIPs, like LDL receptor-mediated endocytosis for saporin (a type I RIP isolated from *Saponaria officinalis* L.), have been described [11,12,13,14]. After endocytosis, type I RIPs accumulate in late endosomes and lysosomes where, for the most part, they are degraded [13,15,16,17]. They escape lysosomal degradation only to a very low degree, resulting in rather low cytotoxicity [9,18,19]. 

The co-application of triterpene saponins, synthesized in the same plants as type I RIPs, significantly increases the cytotoxicity of type I RIPs, as well as the cytotoxicity of type I RIPs conjugated to antibodies, so-called targeted toxins [17,20,21,22]. SO1861, a bisdesmosidic triterpene saponin isolated from the roots of *Saponaria officinalis* L., is one of the cytotoxicity-enhancing saponins for type I RIPs [21].

Co-localization studies have revealed that enhanced cytotoxicity is caused by saponin-mediated endosomal escape from late endosomes/lysosomes [16,17,21]. Membrane-permeabilizing effects at applied concentrations could be excluded [23]. Weng et al. and Bachran et al. reported that the acidic pH of late endosomes/lysosomes is mandatory for endosomal escape enhancement [17,24]. In addition, surface plasmon resonance spectroscopy (SPR) has indicated a direct interaction of triterpene saponins and type I RIPs. The PH-dependent binding of SO1861 and SA1641, a saponin isolated from *Gypsophila paniculata* L., to ^His^saporin, a type I RIP from the seeds of *Saponaria officinalis* L., can be demonstrated by SPR [17,25]. 

Type I RIPs have high potential in the development of targeted toxin-based anti-cancer therapy. Up to the present, however, not a single drug candidate of this type has been approved by a drug agency [26,27]. On the one hand, there have been major medical issues, including immunogenicity and vascular leak syndrome, reported in clinical studies [28,29,30]. On the other hand, the therapeutical use is limited by restricted endosomal escape, which means that high doses would have to be used to achieve an effect. However, high doses are also associated with clinically adverse effects [26]. Thus, insights into the molecular mechanism underlying saponin-triggered endosomal escape may present a valuable contribution toward bringing to fruition the targeted toxin approach for cancer treatment.

To date, little is known about the molecular interaction mechanism underlying endosomal escape enhancement. This is the first study to characterize the involvement of basic amino acid residues in the endosomal escape enhancement induced by triterpene saponins and in the endocytosis of type I RIPs. Therefore, the type I RIP dianthin-30 (~30 kDa) and the triterpene saponin SO1861 were selected for this study. Dianthin-30, located in different tissues of the plant *Dianthus caryophyllus* L. (Caryophyllaceae), is a very well-characterized plant protein that exhibits *N*-glycosylase activity (EC 3.2.2.22) [31,32,33]. The recombinant bacterial expression of dianthin-30 was first realized by Legname et al. [34,35]. The triterpene saponin SO1861, isolated from the roots of *Saponaria officinalis* L. (Caryophyllaceae), whose complete structure has been identified, has proven to be very efficient in enhancing endosomal escape [21,36]. 

In the present work, we illustrate that the alanine substitution of single lysine residues, as well as of multiple (up to three) lysine residues, had no effect on the synergistic cytotoxicity of dianthin and SO1861. The alanine substitution of arginine at position 24, however, reduced synergistic cytotoxicity. Experiments were conducted on three cell lines in order to exclude a cell-line-specific effect. By investigating high toxin concentrations without adding SO1861, we could demonstrate that the reduction in synergistic cytotoxicity was not caused by a decreased interaction between dianthin and SO1861. An endocytosis assay revealed that Arg24 contributes to the endocytosis of dianthin.

## 2. Results

### 2.1. Expression and Purification of ^His^dianthin and Its Mutants

We examined all lysine residues of ^His^dianthin (6xHis-tag dianthin-30) except for Lys138, Lys211 and Lys248. Lys138 and Lys211 are present in active sites and involved in enzymatic activity, while Lys248 electrostatically stabilizes the active site [37]. Additionally, Arg24 was included in the mutational analysis, as Di Maro et al. found that arginine at position 24 is highly conserved in type I and type II RIPs by comparing various RIP sequences [38]. To study the role of the basic residues, lysine and arginine were substituted with alanine. Alanine substitution comes along with no hydrogen bonding; no sterical hindrance; or no insertion of a hydrophobic side chain [39]. Consequently, the side chain is replaced without affecting the conformation of the main chain [40]. A total of 19 ^His^dianthin mutants were generated: Lys50Ala, Lys92Ala, Lys113Ala, Lys126Ala, Lys129Ala, Lys156Ala, Lys157Ala, Lys162Ala, Lys190Ala, Lys195Ala, Lys201Ala, Lys221Ala, Lys227Ala, Lys235Ala, Lys240Ala, Lys254Ala, Lys50/92Ala, Lys50/92/126Ala and Arg24Ala. While the pET11d vectors containing the Lys195Ala, Lys227Ala and Arg24Ala mutations were created by site-directed mutagenesis (see Supplementary Figure S1), the remaining vectors were synthesized externally (BioCat, Heidelberg, Germany). The mutants of ^His^dianthin were mainly expressed in NiCo21(DE3) *E. coli* cells. The expression of some mutants (Lys156Ala, Lys157Ala, Lys235Ala, Lys240Ala and Lys50/92/126Ala) was not possible in NiCo21(DE3) but could be achieved in BL21(DE3)pLysS *E. coli.* The recombinant proteins were purified in small batches by Ni-NTA affinity chromatography using rapid centrifugation-based purification. ^His^dianthin and ^His^dianthin Arg24Ala were additionally prepared in larger batches by combining manual Ni-NTA and cation exchange chromatography. 

**Figure 1:**
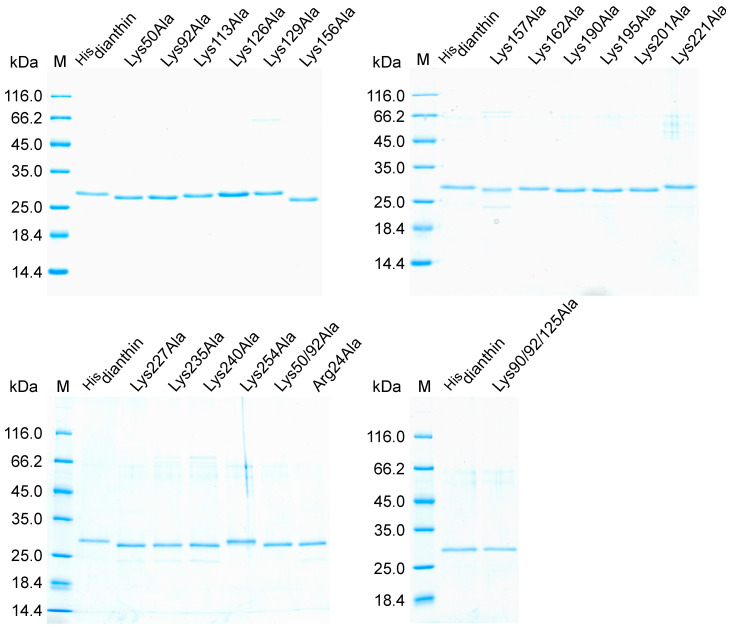
SDS-PAGE (12.5%, Coomassie Brillant Blue stain) analysis of ^His^dianthin and ^His^dianthin mutants. The purified protein fractions obtained after rapid centrifugation-based Ni-NTA purification are visualized. In total, 0.6 µg of protein was applied to each pocket. Protein marker (M, in kDa) and the reference protein ^His^dianthin have the same position in each gel.

SDS-PAGE analysis of recombinantly expressed and purified ^His^dianthin mutants revealed, for all mutants, a main band at approximately the same height as the reference ^His^dianthin (Figure 1). The main bands could, therefore, be assigned to the target proteins. In addition to the target protein band, the purified protein fractions of ^His^dianthin mutants Lys129Ala, Lys157Ala, Lys221Ala, Lys227Ala, Lys235Ala, Lys240Ala, Lys254Ala and Arg24Ala showed further weak protein bands that corresponded to *E. coli* contaminations [41]. 

Due to the high number of proteins to be expressed, entirely pure elution fractions were not used at this point. ^His^dianthin mutants that would show a difference from native ^His^dianthin in cytotoxic screening should be re-expressed and purified to homogeneity. In the end, this occurred only for ^His^dianthin Arg24Ala. 

In addition to SDS-PAGE analysis, ^His^dianthin and ^His^dianthin Arg24Ala obtained by cation exchange chromatography were analyzed using trypsin and AspN in-gel digestion (see Supplementary Figure S2). A comparison of tryptic peptide mass fingerprints allowed us to confirm that the target protein (^His^dianthin Arg24Ala) was derived from ^His^dianthin. To confirm the correct substitution of alanine, the AspN peptide mass fingerprint was evaluated. The peptide at position 21–28 permitted us to differentiate between ^His^dianthin and ^His^dianthin Arg24Ala. The corresponding peptide of ^His^dianthin (DQI**R**NNVR.D) had a mass of 1013.5 Da, while the corresponding peptide of ^His^dianthin Arg24Ala (DQI**A**NNVR.D) shifted by −85 Da to 928.5 Da (see Supplementary Figure S2). The observed mass difference of −85 Da corresponded exactly to the exchange of arginine (156 Da) to alanine (71 Da).

### 2.2. Binding of SO1861 to ^His^dianthin by Native Mass Spectrometry

**Figure 2:**
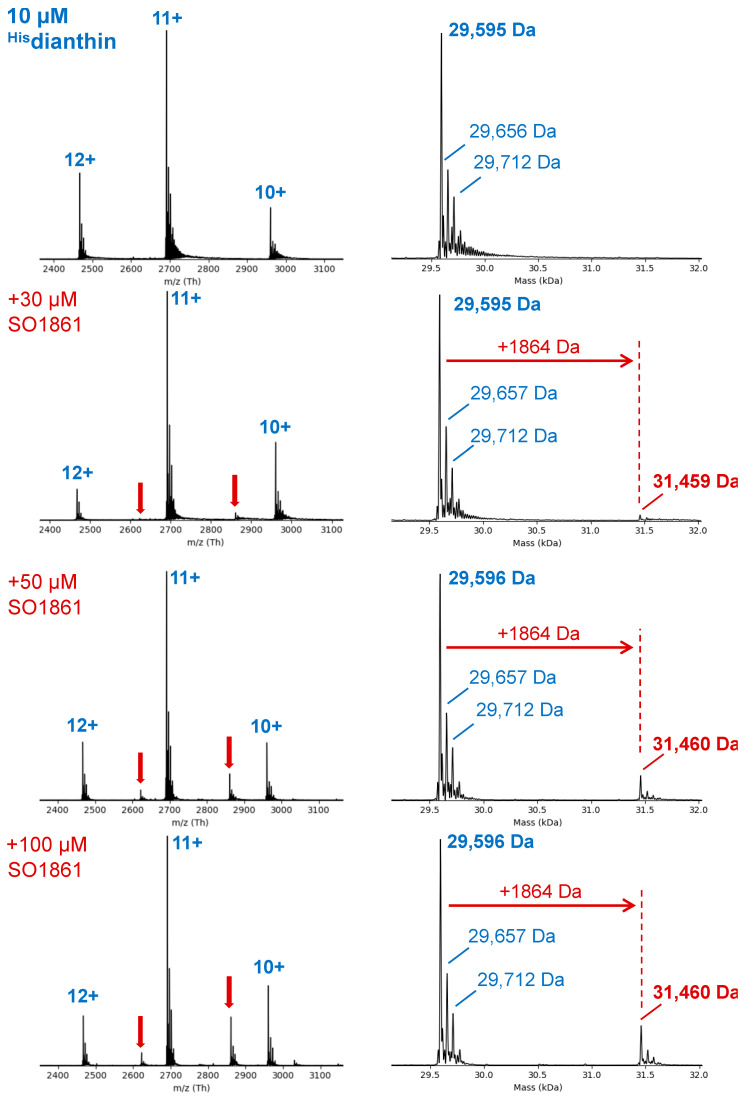
Native MS analysis to study the binding of SO1861 to ^His^dianthin. Samples were dissolved in 50 mM of ammonium acetate buffer (pH 6.24), and spectra were recorded with a constant protein concentration (^His^dianthin, 10 µM) and an increasing ligand concentration of SO1861 (0 to 100 µM) from top to bottom. Raw spectra are shown on the left, and corresponding deconvoluted spectra showing the experimental average protein masses are shown on the right. With the addition of SO1861, weak additional signals (highlighted by red arrows) indicate the binding of SO1861 to ^His^dianthin in a 1:1 stoichiometry.

The binding of RIPs and triterpene saponins has, so far, only been demonstrated by SPR for ^His^saporin and the triterpene saponins SO1861 and SA1641 [17,25]. In order to be able to carry out the mutational analysis of ^His^dianthin and to investigate the interaction between SO1861 and ^His^dianthin, we first had to prove that the binding of SO1861 also occurs with ^His^dianthin. To circumvent the drawbacks of SPR measurements of surface-active compounds (unspecific binding to the sensor surface) such as triterpene saponins, native mass spectrometry (MS) was chosen, which detects binding in the gas phase under non-denaturing conditions [42,43]. The native MS measurement of ^His^dianthin without SO1861 showed a typical spectrum for a folded protein, indicated by a low charge state and a narrow charge state distribution ranging from 10+ to 12+ (Figure 2). Using spectral deconvolution, an experimental mass of 29,595 Da was determined for ^His^dianthin. The same mass was confirmed when ^His^dianthin was measured under denaturing MS conditions (see Supplementary Figure S3). For binding studies, ^His^dianthin was analyzed by native MS in the presence of increasing concentrations of SO1861 (3×, 5× and 10× molar excess) were analyzed by native MS (Figure 2). Additional signals at 31,459 Da (+1864 Da) were detected in a concentration-dependent manner that indicated the weak binding of SO1861 to ^His^dianthin in a 1:1 stoichiometry (theoretical MW of SO1861: 1862.9 Da). In addition, no binding or multiple binding could be detected at SO1861 concentrations below 30 µM.

### 2.3. Enzymatic Activities and Cytotoxic Screening of ^His^dianthin Mutants

**Figure 3:**
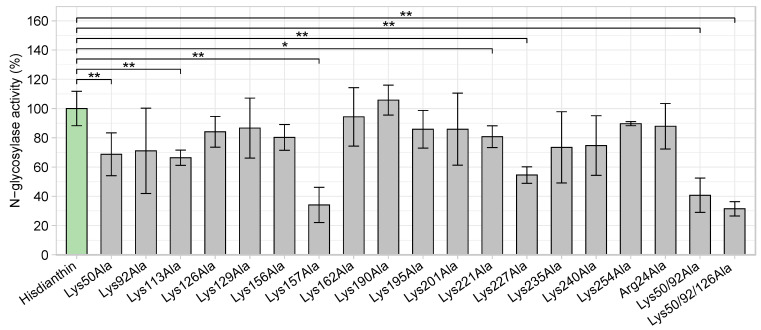
*N*-glycosylase activity of ^His^dianthin mutants in comparison with native ^His^dianthin. The adenine-releasing assay was performed with a protein concentration of 169 nM and 21.4 µM of A30 substrate overnight at 37 °C. The activities of ^His^dianthin mutants were calculated as percentages related to the activity of ^His^dianthin. Lysine substitution at positions 50, 113, 157, 221 and 227 and combined alanine substitutions at positions 50/92 and 50/92/126 significantly reduced the *N*-glycosylase activity. Values represent the means ± standard deviation of three independent measurements; n = 3 (significance: * *p* ≤ 0.05; ** *p* ≤ 0.01; Mann–Whitney *U* test).

An oligonucleotide-based *N*-glycosylase activity assay was used to evaluate the effect of the substitution of different basic residues with alanine on the enzymatic activity of ^His^dianthin [44]. To this end, an artificial oligonucleotide 5′-A_30_-3′ substrate was incubated with native ^His^dianthin (reference) and ^His^dianthin mutants, and the released adenine was quantified by TLC chromatography. The adenine release of native ^His^dianthin was set as the reference value, and the adenine releases of ^His^dianthin mutants were related to it. As shown in Figure 3, all ^His^dianthin mutants were able to release adenine from A30. The single alanine substitutions at positions 50, 113, 157, 221 and 227 caused a significant loss in *N*-glycosylase activity compared with native ^His^dianthin. The *N*-glycosylase activity of ^His^dianthin mutants with multiple alanine substitutions was likewise strongly reduced.

To determine the effect that the alanine substitutions have on the interaction between ^His^dianthin and SO1861, the cytotoxicity of ^His^dianthin mutants in the presence of SO1861 (^His^dianthin mutant + SO1861) was evaluated. To monitor changes in cytotoxicity, a cytotoxic screening was performed with the lowest ^His^dianthin concentration (1 nM) that, in combination with SO1861, still showed full cytotoxicity. In contrast to the native MS measurements, an SO1861 concentration of 1 µg/mL (corresponding to 537 nM) was used for the cytotoxicity experiments. Applicable SO1861 concentrations are limited by the cytotoxic effect of triterpene saponins. For SO1861, cytotoxicity was detected in Neuro-2A cells at concentrations of 7.5 µg/mL (corresponding to 4 µM) and above [45]. Previous studies have shown that SO1861 concentrations of 1 or 2 µg/mL are sufficient to enable efficient endosomal escape [16,46].

**Figure 4:**
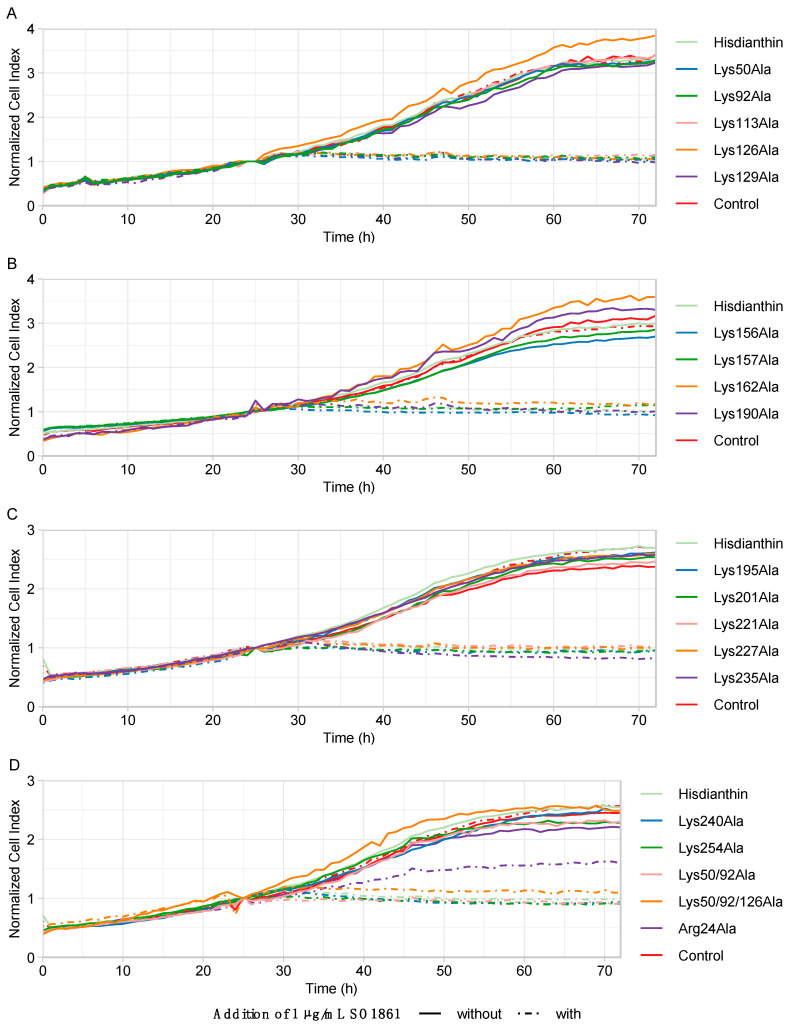
Cytotoxic screening of ^His^dianthin mutants in Neuro-2A cells. (**A**) ^His^dianthin mutants Lys50Ala to Lys129Ala. (**B**) ^His^dianthin mutants Lys156Ala to Lys190Ala. (**C**) ^His^dianthin mutants Lys195Ala to Lys235Ala. (**D**) ^His^dianthin mutants Lys240Ala to Arg24Ala. Cytotoxicity is represented using cell growth data. The cell confluence is shown as normalized cell index, which was calculated by dividing the cell confluence at each time point by the cell confluence at the reference time point of 25 h (time point after sample addition). In total, cell confluence was recorded over 72 h using the live-cell imaging CytoSMART Omni system. Then, 24 h after seeding the cells, 1 nM of ^His^dianthin mutant either with the addition of 1 µg/mL of SO1861 or without the addition of SO1861 (1 nM ^His^dianthin mutant ± 1 µg/mL SO1861) was added and incubated for 48 h. Control cells were treated the same way. As a positive control, 1 nM of native ^His^dianthin ± 1 µg/mL SO1861 was used, and as a negative control, PBS ± 1 µg/mL SO1861 was used. Samples without the addition of SO1861 should have no effect on cell growth. The addition of SO1861 should reveal the cytotoxic effect of the ^His^dianthin mutants and the ^His^dianthin control. The samples without the addition of SO1861 are shown as solid lines, while the samples with the addition of SO1861 are shown as dashed lines. Data are shown as means of three independent measurements, each in triplicate (n = 3). For clarity, the standard deviation is not shown.

Alanine substitutions affecting the molecular interaction between ^His^dianthin and SO1861 would consequently escape less from the endosomes and, therefore, reduce cytotoxicity. As shown in Figure 4, only 1 of 19 alanine substitutions showed reduced synergistic cytotoxicity. ^His^dianthin Arg24Ala was the only mutant that behaved differently from the native ^His^dianthin and was, therefore, the only ^His^dianthin mutant subjected to further characterizing experiments.

### 2.4. Cytotoxic Characterization of ^His^dianthin Arg24Ala

**Figure 5:**
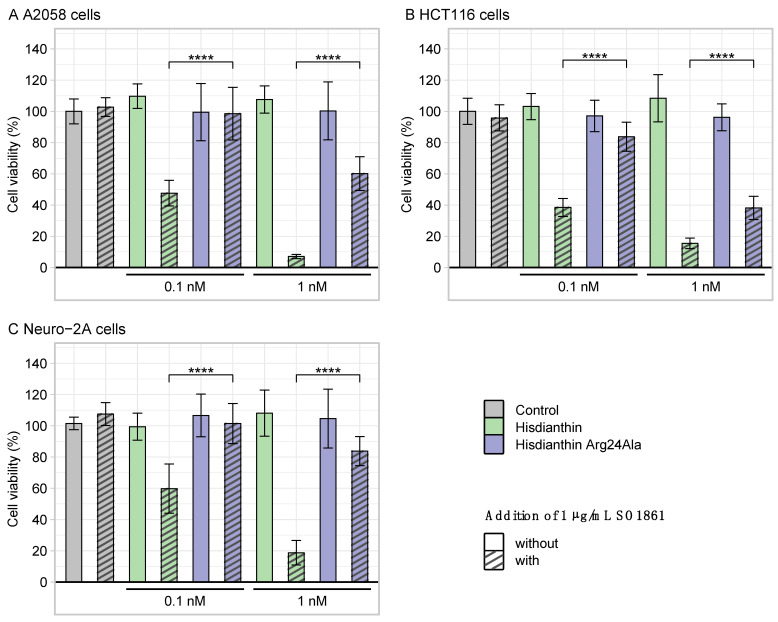
Cytotoxicity of ^His^dianthin Arg24Ala in three cell lines. A2058 cells (**A**), HCT116 cells (**B**), and Neuro-2A cells (**C**) were incubated with 0.1 nM and 1 nM of ^His^dianthin and ^His^dianthin Arg24Ala ± 1 µg/mL of SO1861 for 48 h, respectively. Control cells were equivalently treated with PBS ± 1 µg/mL of SO1861. After 48 h of incubation, an MTT assay was performed. The presented cell viability was calculated relative to the control cells. The Arg24Ala mutation caused significantly reduced synergistic cytotoxicity (+SO1861) in all cell lines compared with native ^His^dianthin. Data are shown as means ± standard deviation of three independent measurements, each in triplicate; n = 3 (Significance: **** *p* ≤ 0.0001, Student’s *t*-test).

As confluence data for the ^His^dianthin Arg24Ala in Neuro-2A cells (Figure 4) suggested a reduction in synergistic cytotoxicity compared with native ^His^dianthin, the significance of reduced cytotoxicity and cell line specificity was investigated. To exclude that the reduction in cytotoxicity was caused by a Neuro-2A-cell-line-specific effect, ^His^dianthin Arg24Ala (±SO1861) was also tested in A2058 and HCT116 cells. Cytotoxicity was measured by means of cell viability. As shown in Figure 5, the synergistic cytotoxicity of 0.1 nM, as well as 1 nM ^His^dianthin Arg24Ala, was significantly reduced in all cell lines studied. 

**Figure 6:**
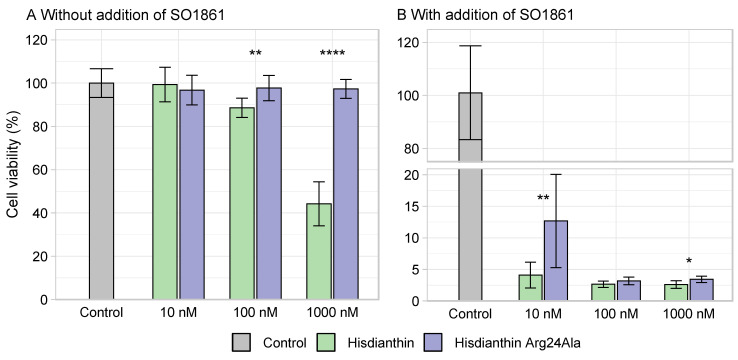
Cytotoxicity of high concentrations of ^His^dianthin Arg24Ala. Neuro-2A cells were incubated with either 10 nM, 100 nM or 1000 nM ^His^dianthin and ^His^dianthin Arg24Ala without the addition of SO1861 (**A**) and with the addition of 1 µg/mL of SO1861 (**B**) for 48 h. Control cells were treated equivalently with PBS ± 1 µg/mL of SO1861. At the end of incubation, an MTT assay was performed. (**A**) Without the addition of SO1861, ^His^dianthin Arg24Ala was not cytotoxic even at high concentrations, whereas native ^His^dianthin showed cytotoxicity above a concentration of 100 nM, even without the addition of SO1861. The cytotoxicity of the native and mutant forms differed significantly above a concentration of 100 nM. Data are shown as means ± standard deviation of three independent measurements, each in triplicate; n = 3 (significance: ** *p* ≤ 0.01; **** *p* ≤ 0.0001; Student’s *t*-test). (**B**) the addition of SO1861 caused an increase in cytotoxicity in both forms. The cytotoxicity differences in both forms were significant at 10 nM and at 1000 nM. Data are shown as means ± standard deviation of three independent measurements, each in triplicate; n = 3 (significance: * *p* ≤ 0.05; ** *p* ≤ 0.01; Mann–Whitney *U* test).

To determine whether the loss of cytotoxicity observed in ^His^dianthin Arg24Ala was due to a reduced interaction with SO1861, its cytotoxicity was measured at high concentrations with and without the addition of SO1861. ^His^dianthin escapes lysosomal degradation in very small amounts through a mechanism that has not been elucidated completely. High toxin concentrations allow us to measure the cytotoxic effect of RIPs, even without the addition of triterpene saponins. The higher the RIP concentrations, the higher the amount of protein that is able to reach the cytosol and exert its cytotoxic effect [13,18]. As shown in Figure 6A, while ^His^dianthin was cytotoxic at concentrations above 100 nM, even without the addition of SO1861, the insertion of the Arg24Ala mutation caused a complete loss of cytotoxicity. Also, at high concentrations, with the addition of SO1861, the cytotoxicity of ^His^dianthin Arg24Ala stayed slightly reduced compared with native ^His^dianthin (Figure 6B). The synergistic cytotoxicity of ^His^dianthin Arg24Ala (+SO1861) at lower concentrations (0.1 nM, 1 nM and 10 nM) was significantly reduced or absent compared with native ^His^dianthin. At the same time, the cytotoxicity of ^His^dianthin Arg24Ala at higher concentrations (100 nM and 1000 nM) without the addition of SO1861 (–SO1861) was also reduced. Consequently, the reduced cytotoxicity could not be caused by decreased interactions with SO1861. Reduced cytotoxicity due to decreased *N*-glycosylase activity could also be excluded, as the enzyme activity of ^His^dianthin Arg24Ala and ^His^dianthin did not differ significantly (Figure 3). Since the reduced cytotoxicity of ^His^dianthin Arg24Ala could be attributed neither to a decreased interaction with SO1861 nor to a loss of enzyme activity, it was hypothesized that it was caused by the reduced endocytosis of ^His^dianthin Arg24Ala.

### 2.5. Characterization of Labeled Proteins—^His^dianthin-CF568 and ^His^dianthin Arg24Ala-CF568

**Figure 7:**
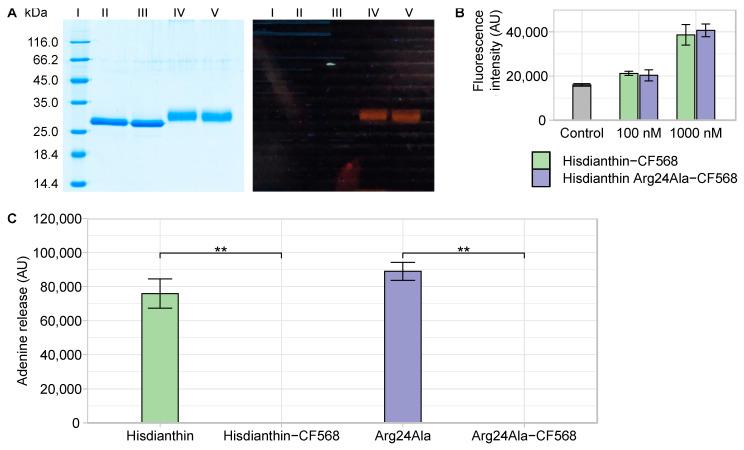
Characterization of ^His^dianthin-CF568 and ^His^dianthin Arg24Ala-CF568. (**A**) SDS-PAGE (12.5%, Coomassie Brillant Blue stain) of the labeling reaction of ^His^dianthin und ^His^dianthin Arg24Ala with CF^®^568 resulted in clean labeling products (each one a diffuse band at 30–33 kDa). Equivalent section: Left side is shown under daylight and right side at 366 nm. Lane I: Protein marker (in kDa). Lane II: ^His^dianthin (2.0 µg). Lane III: ^His^dianthin Arg24Ala (2.0 µg). Lane IV: ^His^dianthin-CF568 (2.3 µg). Lane V: ^His^dianthin Arg24Ala-CF568 (2.3 µg). (**B**) Fluorescence intensity of ^His^dianthin-CF568 and ^His^dianthin Arg24Ala-CF568 at 560/10 nm (excitation wavelength) and 595/35 nm (emission wavelength). No significant difference in intensity was measured at 100 nM or at 1000 nM of ^His^dianthin-CF568 and ^His^dianthin Arg24Ala-CF568. PBS was measured as a control. Data are shown as means ± standard deviation of three independent measurements, each in triplicate; n = 3 (significance: Mann–Whitney *U* test). (**C**) *N*-glycosylase activity of ^His^dianthin-CF568 and ^His^dianthin Arg24Ala-CF568. The adenine-releasing assay was performed with a protein concentration of 169 nM and 21.4 µM of A30 substrate at 37 °C overnight. Labeling with fluorescent dye CF^®^568 resulted in complete loss of activity in ^His^dianthin-CF568 and ^His^dianthin Arg24Ala-CF568. The means ± standard deviation of three independent measurements are shown; n = 3 (significance: ** *p* ≤ 0.01; Student’s *t*-test).

In order to test the hypothesis that the Arg24Ala mutation affects the endocytosis of ^His^dianthin, an endocytosis assay was performed. For this purpose, ^His^dianthin and ^His^dianthin Arg24Ala were labeled with CF^®^568, a red fluorescence label. Consequently, labeled ^His^dianthin and ^His^dianthin Arg24Ala were characterized (Figure 7). 

^His^dianthin and ^His^dianthin Arg24Ala reacted completely to ^His^dianthin-CF568 and ^His^dianthin Arg24Ala-CF568 (Figure 7A, lanes IV and V). Each labeled product resulted in only one band mass in a range of about 30–33 kDa, which was slightly diffuse, caused by different labeling degrees. By measuring the absorption at 280 nm and 562 nm, the degree of labeling (DOL) was determined as 2.1 for ^His^dianthin-CF568 and 2.4 for ^His^dianthin Arg24Ala-CF568. An additional MALDI-TOF analysis confirmed the purity of the labeled proteins and could identify the range of the DOL, which was between 2 and 6 for ^His^dianthin-CF568 and between 1 and 6 for ^His^dianthin Arg24Ala-CF568 (see Supplementary Figure S4). As shown in Figure 7B, the fluorescence intensity of ^His^dianthin-CF568 and ^His^dianthin Arg24Ala-CF568 differed neither at 100 nM nor at 1000 nM. Thus, the slightly varying DOL had no effect on fluorescence intensity. The CF^®^568-labeled proteins ^His^dianthin and ^His^dianthin Arg24Ala completely lost their oligonucleotide *N*-glycosylase activity (Figure 7C). In addition, the cytotoxicity assay revealed that ^His^dianthin Arg24Ala-CF568 (+SO1861) did not show any cytotoxic effect in the concentration range studied from 0.1 nM to 100 nM. ^His^dianthin-CF568 (+SO1861) remained cytotoxic only at 10 nM (see Supplementary Figure S5).

### 2.6. Endocytosis of ^His^dianthin-CF568 and ^His^dianthin Arg24Ala-CF568

**Figure 8:**
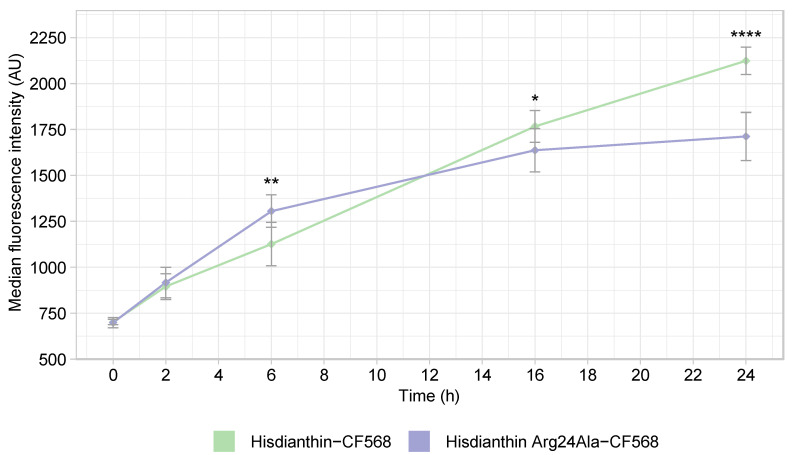
Endocytosis of ^His^dianthin-CF568 and ^His^dianthin Arg24Ala-CF568. Neuro-2A cells were incubated for 0, 2, 6, 16 and 24 h with 100 nM of ^His^dianthin-CF568 and ^His^dianthin Arg24Ala-CF568. After incubation with a labeled protein, the cell culture medium was removed, and cells were analyzed by flow cytometry using the peak height of the PE channel. Endocytosis was measured by means of fluorescence intensity increase. After 6 h, ^His^dianthin-CF568 and ^His^dianthin Arg24Ala-CF568 differed significantly in their endocytosis, where, first, ^His^dianthin Arg24Ala-CF568 and, after 16 h, ^His^dianthin-CF568 were more endocytosed. At least 10,000 cells per sample were measured. Only single cells were included in the analysis; doublets and debris were excluded. The means ± standard deviation of three independent measurements are shown; n = 3 (significance: * *p* ≤ 0.05; ** *p* ≤ 0.01; **** *p* ≤ 0.0001; Student’s *t*-test).

Flow cytometry was used to quantify the endocytosis of fluorescence-labeled ^His^dianthin and ^His^dianthin Arg24Ala into Neuro-2A cells. Therefore, cells were incubated with a labeled protein over a time course of 24 h and evaluated by flow cytometry via the peak height of the PE channel. The extent of the endocytosis of the labeled protein was proportional to the peak height of the fluorescence signal. At time 0 h, no endocytosis had occurred. The fluorescence intensity at this time point corresponded to the intrinsic fluorescence of the cells. As shown in Figure 8, during the first two hours, the extent of the endocytosis of ^His^dianthin-CF568 and ^His^dianthin Arg24Ala-CF568 was essentially identical. After 6 h, ^His^dianthin Arg24Ala-CF568 was endocytosed to a significantly higher proportion than ^His^dianthin-CF568, whereas, after 16 h, the endocytosis of ^His^dianthin-CF568 exceeded that of ^His^dianthin Arg24Ala-CF568. After 24 h, the uptake of ^His^dianthin-CF568 into the cell remained significantly higher than that of ^His^dianthin Arg24Ala-CF568. This experiment confirms the hypothesis that the reduced synergistic cytotoxicity of ^His^dianthin Arg24Ala was due to lower endocytosis compared with native ^His^dianthin. Arginine at position 24 thus seems to play an important role in the endocytosis of ^His^dianthin.

## 3. Discussion

Synergistic cytotoxicity between type I RIPs and triterpene saponins was first demonstrated by Hebestreit and Melzig in 2003 for agrostin and agrostemmasaponins, both isolated from the seeds of *Agrostemma githago* L. [20]. In recent years, it has also been shown that the cytotoxicity of other type I RIPs, such as dianthin-30 and saporin-S6, as well as various targeted toxins, is also enhanced by triterpene saponins [16,17]. The enhanced cytotoxicity is due to an endosomal release of type I RIPs mediated by saponins [22,47]. 

This study was undertaken in order to investigate the exact molecular mechanism of this process. In order to be effective, triterpene saponins must fulfill specific structural requirements; the presence of a glucuronic acid at position C-3 is one of these [23,48,49,50]. At the same time, acidic pH in late endosomes (pH 5.0–5.5) and lysosomes (pH 4.5–5.0) plays an important role in endosomal escape enhancement [17,24,51]. 

This study focuses on ^His^dianthin, a very well-characterized type I RIP, and SO1861 [21,32]. Considering the theoretical isoelectric point (IEP) of ^His^dianthin at 9.48, the protein or much more its basic residues are positively charged under the above-mentioned acidic pH conditions, whereas the glucuronic acid of SO1861 is negatively charged. In previous studies, the binding of triterpene saponins to type I RIPs showed pH-dependency, with the strongest binding at pH 5, which is in accordance with the importance of acidic pH in endosomes and lysosomes [17,25]. Based on these findings, we assumed that basic amino acids can interact with the glucuronic acid of SO1861. The aim was to study all lysine residues that were surface-accessible and not localized in the active site. Residues involved in rRNA *N*-glycosylase activity were excluded from the analysis. The adenine-releasing activity of ^His^saporin in the presence of SA1641 did not show any significant reduction in activity, indicating that triterpene saponins do not bind to the active center [52]. 

A total of 19 ^His^dianthin mutants were generated: 1 arginine mutant, 15 single lysine mutants, 1 double lysine mutant and 1 triple lysine mutant. In addition to the single substitutions, we decided to include double and triple substitutions to investigate the effect of the IEP of ^His^dianthin on its interaction with SO1861. Single lysine substitutions led to a reduction in the theoretic IEP, from 9.48 to 9.42, whereas double and triple lysine substitutions reduced the theoretic IEP to 9.35 and 9.26, respectively [53].

As mentioned earlier, Weng et al. investigated the binding of type I RIPs and triterpene saponins using the example of ^His^saporin and SO1861/SA1641 with SPR [17,25]. So far, binding data for other type I RIPs and triterpene saponins are not available. We performed native mass spectrometry in order to demonstrate the binding of SO1861 to ^His^dianthin. The confirmation of binding between ^His^dianthin and SO1861 was critical for the subsequent mutational analysis. 

As shown in Figure 2, with increasing concentrations of SO1861, an additional small binding signal was detected. Since only one binding signal occurred, a 1:1 stoichiometry can be assumed. The experiment was performed with molar excesses of 3×, 5× and 10× SO1861. The SO1861 concentrations used for native MS measurements (Figure 2) differed from the concentrations used during cell culture experiments (Figure 4, Figure 5 and Figure 6). The binding signal could only be detected at an SO1861 concentration greater than 30 µM, which indicates low-affinity binding between ^His^dianthin and SO1861. Accordingly, the SO1861 concentration of 1 µg/mL (corresponding to 537 nM) was unsuitable for native MS measurements. Equally, SO1861 concentrations of 7.5 µg/mL (corresponding to 4 µM) or more impair cell viability, even without combination with type I RIPs [45]. Therefore, SO1861 concentrations in cell culture experiments to study the synergistic cytotoxicity of saponins and type I RIPs are limited by their own cytotoxicity. An SO1861 concentration of 1 µg/mL was chosen, as this concentration was shown to be as efficient in enhancing endosomal escape as 2 µg/mL [16,46].

While Weng et al. reported the binding of SO1861 and SA1641 to ^His^saporin using SPR, we could prove the binding of SO1861 to ^His^dianthin by native MS. Thus, the binding of SO1861 to two distinct type I RIPs, ^His^saporin and ^His^dianthin, using two strongly differing methods (SPR and native MS), has been demonstrated. Both methods have weaknesses. In SPR, ligands can bind unspecifically to the sensor surface, which can be very pronounced for surface-active molecules such as SO1861, and the immobilization on the sensor surface can cause changes in native protein conformation [42]. In native MS, high ligand concentrations can also cause unspecific binding, which usually appears as multiple binding. For SO1861, no multiple binding could be detected. The results reported by Weng et al. and our results suggest that triterpene saponins bind to type I RIPs [17,25].

This is the first study reporting a mutational analysis of ^His^dianthin. So far, the mutational data for type I RIPs with similar sequences have only been available for ^His^saporin, but these studies mainly focused on active-site residues [54,55,56]. No data are available regarding how the substitution of lysine with alanine at specific positions affects *N*-glycosylase activity and cytotoxicity, with the exception of the mutation Arg24Ala, which was studied using the example of ^His^saporin. ^His^saporin shares 79.8% sequence identity with ^His^dianthin. This level of sequence homology is comparatively high and allows for robust inferences from one protein to the other [32]. In addition, the main catalytic residues, Tyr73, Glu177 and Arg180 (dianthin-30 numbering), which are highly conserved in the RIP plant toxin family (applicable to type I and type II RIPs), have the same localization as Arg24 in ^His^dianthin, as well as in ^His^saporin [37,57].

Alanine substitution at position 24 did not affect ^His^saporin’s enzymatic activity or its protein synthesis inhibitory activity. In this case, *N*-glycosylase activity was determined by measuring the release of an Endo fragment from the 28S rRNA of rabbit reticulocyte lysate and protein synthesis inhibition using an in vitro translational assay [55]. The release of the Endo fragment from rabbit reticulocyte lysate performed by Bagga et al. more accurately mimics the cellular processes than the adenine-releasing assay [44,55]. Since ^His^saporin and ^His^dianthin are strongly consistent in their sequence (79.8% sequence identity) and no change in adenine-releasing activity was observed for ^His^dianthin Arg24Ala, it can be assumed that the substitution of the residue Arg24 by alanine should have the same effect on the protein synthesis inhibitory activity of ^His^dianthin, as demonstrated for ^His^saporin Arg24Ala by Bagga et al. [55].

As shown in Figure 3 and Figure 4, adenine-releasing activity is not correlated with the extent of cytotoxicity. The insertion of the alanine substitution at positions 50, 113, 157, 221, 227, 50/92 and 50/92/126 significantly reduced their *N*-glycosylase activity, but their synergistic cytotoxicity was not affected. Even though the lysines at these positions are not involved in the enzymatic reaction mechanism of ^His^dianthin, their *N*-glycosylase activity was reduced [37]. The reduction was probably due to a global destabilizing effect that the insertion of alanine substitutions may have on the protein structure [58,59]. 

Cytotoxic screening was used to study the interaction between ^His^dianthin mutants and SO1861. Reduced interaction between the ^His^dianthin mutant and SO1861 would mean less endosomal escape and, consequently, a reduction in synergistic cytotoxicity. As previously mentioned, the SO1861 concentration (1 µg/mL) and the protein concentration (1 nM) were optimized in order to determine the smallest change in synergistic cytotoxicity caused by the mutation. 

None of the lysine mutants showed any reduction in synergistic cytotoxicity. The studied lysine residues thus had no effect on the interaction with SO1861 and were not involved in the molecular mechanism of endosomal escape enhancement. Furthermore, the reduction in the IEP from 9.48 to 9.26 had no effect on the interaction between ^His^dianthin and SO1861. Hebestreit et al. supposed Schiff base formation between the aldehyde function of the aglycone and specific amino acids like lysines [60]. Böttger et al. already questioned this hypothesis, as they tested saponins with aldehyde function and could not detect any enhanced endosomal release [23]. Our data also contradict the theory of Schiff base formation with lysines as the mechanism. None of the tested lysine mutants impacted the synergistic cytotoxicity. 

**Figure 9:**
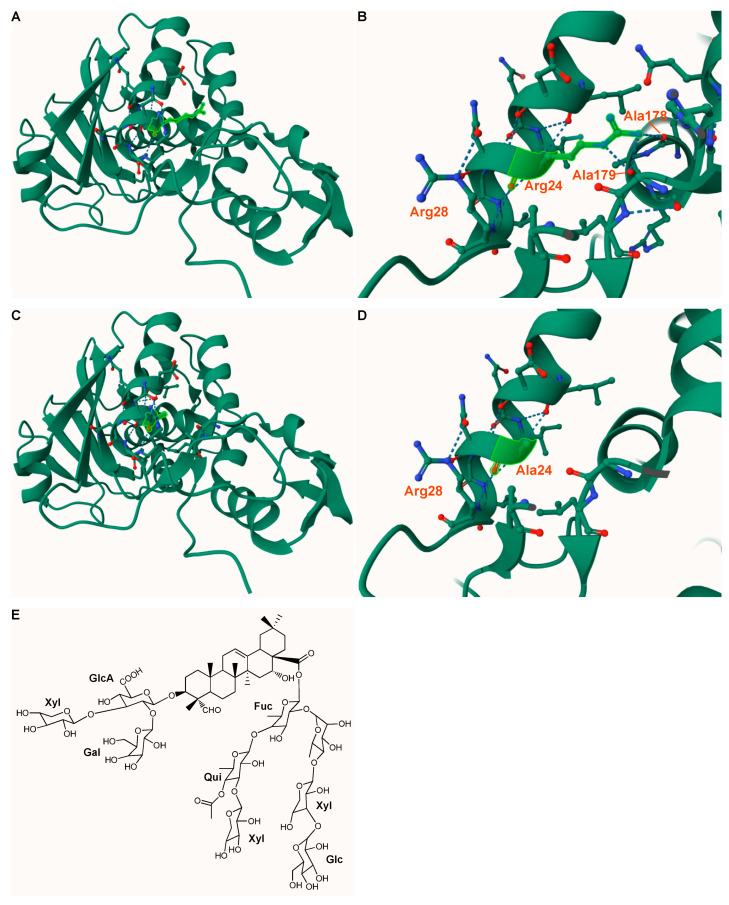
Protein structure of ^His^dianthin and its mutant ^His^dianthin Arg24Ala and structure of SO1861. (**A**–**D**) Homology model of ^His^dianthin and ^His^dianthin Arg24Ala using Phyre^2^ and Mol* Viewer [63,64]. The high-resolution structure of dianthin-30 served as template (1.4 Å; PDB ID 1RL0) [65]. The homology models indicate confidence and coverage levels of 100%. (**A**,**B**) Tertiary structure of ^His^dianthin. The residue Arg24 (highlighted in light green) is surface-accessible and interacts via hydrogen bonds with the residues Arg28, Ala179 and Ala178. (**C**,**D**) Tertiary structure of ^His^dianthin Arg24Ala. After inserting an alanine at position 24, the hydrogen bonds with Ala178 and Ala179 no longer exist. Ala24 now interacts only with Arg28. (**E**) Structure of the bisdesmosidic triterpene saponin SO1861 [36].

^His^dianthin Arg24Ala was the only ^His^dianthin mutant whose synergistic cytotoxicity was altered by the insertion of the mutation (Figure 4). This appears to be a general rather than cell-type-specific phenomenon since it was similarly observed in three different cell lines. Arg24 would be a suitable candidate as an interaction partner with SO1861. As shown in Figure 9, the amino acid is surface-accessible and is surrounded by hydrophilic and hydrophobic residues, matching the amphiphilic structure of SO1861 [61]. 

To confirm that the reduced combinatorial cytotoxicity is based on the lower interaction between ^His^dianthin Arg24Ala and SO1861, ^His^dianthin Arg24Ala was studied at high protein concentrations, allowing us to investigate cytotoxicity without the addition of SO1861 [32,62]. Since the cytotoxicity of ^His^dianthin and ^His^dianthin Arg24Ala varied even without the addition of SO1861, the reduced synergistic cytotoxicity could not be due to a reduced interaction with SO1861. As also shown for ^His^saporin Arg24Ala, the insertion of Arg24Ala in ^His^dianthin had no impact on its *N*-glycosylase activity [55]. Consequently, the reduced cytotoxicity could only have been caused by a lower toxin concentration in the cytosol. This decreased concentration, however, is not due to a reduced endosomal release but to a reduced uptake into the cell by endocytosis.

The fluorescent labeling of type I RIPs has been described for ^His^dianthin, ^His^saporin and a saporin-based immunotoxin [11,15,17,47,66]. None of these authors have reported DOL or effects on *N*-glycosylase activity. No information on the number of bound fluorescent molecules to type I RIPs is available to evaluate our DOL data. Labeled ^His^saporin remained fully cytotoxic [11]. The determined DOL of ^His^dianthin-CF568 and ^His^dianthin Arg24Ala-CF568, each 2.1 and 2.4, respectively, deviated slightly from the manufacturer’s recommendations, which were 1.0–1.2. The unspecific binding of CF^®^568 to the primary amines of lysine residues caused a loss of *N*-glycosylase activity and cytotoxicity at low toxin concentrations. To avoid unspecific binding, a more selective labeling technique could be performed. Thiol-reactive reagents would be more selective, as there is only one cysteine in ^His^dianthin. The unique cysteine of ^His^dianthin, however, is not surface-accessible and, therefore, not suitable for thiol labeling [61,67]. 

The uniformity of the DOL of ^His^dianthin-CF568 and ^His^dianthin Arg24Ala-CF568 could be confirmed with both SDS-PAGE and MS (Figure 7 and Figure S4). The fluorescence intensity of both proteins was also the same. Assuming that both proteins were labeled with CF568 at the same lysine residues, ^His^dianthin-CF568 and ^His^dianthin Arg24Ala-CF568 fulfilled all criteria for comparing their endocytosis. In addition, there was no lysine residue in the vicinity of Arg24, which, by binding to CF^®^568, would sterically hinder the accessibility of the arginine. Within 24 h, more ^His^dianthin-CF568 accumulated in the cells than ^His^dianthin Arg24Ala-CF568 (Figure 8). Under the assumptions made, our data suggest that Arg24 may make a functional contribution to the endocytosis of ^His^dianthin. Considering the tertiary structure of ^His^dianthin (Figure 9), the position of Arg24 allows it to be accessed from the surface and makes it conducive to endocytosis. 

Arginine at position 24 is not only preserved in dianthin-30 but also in saporin-S6, as previously shown [55]. In addition, the alignment of dianthin-30 with common type I and type II RIPs indicates that Arg24 is also conserved in sapovaccarin-S1, gelonin, gypsophilin-S, agrostin, bouganin, abrin A chain and ricin A chain [34,46,68,69,70,71,72,73]. Thus, Arg24 is even found in type I RIPs like agrostin, which have an amino acid sequence that is unusual for the latter ones. Moreover, Arg24 is even preserved in type II RIPs, whose endocytosis mechanism is demonstrably different from that of type I RIPs [9]. The presence of arginine at the same position suggests that the role of Arg24 in the endocytosis of dianthin-30 is also transferable to other type I RIPs. However, it remains questionable whether Arg24 has the same function during endocytosis in type I and type II RIPs.

## 4. Conclusions

Nineteen ^His^dianthin mutants were analyzed for their interactions with SO1861 on the basis of their synergistic cytotoxicity. None of the ^His^dianthin mutants studied affected the endosomal escape enhancement induced by triterpene saponins. According to our findings, lysine residues and Arg24 are not involved in the interaction between ^His^dianthin and SO1861. The reduced synergistic cytotoxicity of ^His^dianthin Arg24Ala could be attributed to a lower extent of endocytosis. It took 20 years to elucidate parts of the endosomal escape enhancement mechanism. This study was also unable to unveil the complete mechanism underlying the enhancement of endosomal escape. However, it succeeded in defining a number of residues that are clearly not involved in this mechanism.

## 5. Materials and Methods

### 5.1. Construction of RIP-Mutants

The nucleotide sequence of dianthin-30 tagged with an *N*-terminal 6x polyhistidine-tag (referred to as ^His^dianthin) was present in a pET11d vector [16]. The 6114-base pair pET11d-^His^dianthin vector was used as a template to mutate the codons for basic amino acid residues Lys195, Lys227 and Arg24 to that for alanine via site-directed mutagenesis. The mutations were inserted using the Q5^®^ Site-Directed Mutagenesis Kit (New England Biolabs, Ipswich, MA, USA) following the manufacturer’s instructions. DNA primers containing the mutations Lys195Ala, Lys227Ala and Arg24Ala were designed according to NEBaseChanger^®^ (New England Biolabs, Ipswich, MA, USA; Table 1). After performing PCR, products were visualized by agarose gel electrophoresis, as described elsewhere [74]. Plasmid preparations (Monarch^®^ Plasmid Miniprep Kit, New England Biolabs, Ipswich, MA, USA) of the resulting mutated vectors and subsequent Sanger DNA sequencing (LGC Genomics, Berlin, Germany) confirmed all mutations. The remaining pET11d-^His^dianthin mutants, Lys50Ala, Lys92Ala, Lys113Ala, Lys126Ala, Lys129Ala, Lys156Ala, Lys157Ala, Lys162Ala, Lys190Ala, Lys201Ala, Lys221Ala, Lys235Ala, Lys240Ala Lys254Ala, Lys50/92Ala and Lys50/92/126Ala, were synthesized by BioCat (Heidelberg, Germany).

### 5.2. Expression and Purification of Recombinant Proteins

^His^dianthin and its mutants Lys50Ala, Lys92Ala, Lys113Ala, Lys126Ala, Lys129Ala, Lys162Ala, Lys190Ala, Lys195Ala, Lys201Ala, Lys221Ala, Lys227Ala, Lys254Ala, Lys50/92Ala and Arg24Ala were recombinantly expressed in *E. coli* NiCo21(DE3) (New England Biolabs, Ipswich, MA, USA). Lys156Ala, Lys157Ala, Lys235Ala, Lys240Ala and Lys50/92/126Ala could not be expressed in NiCo21(DE3). Instead, the BL21(DE3)pLysS strain was used for their expression (Merck Millipore, Burlington, MA, USA). The protein expression followed a protocol described elsewhere [62]. All ^His^dianthin mutants were purified in a small batch by Ni-nitrilotriacetic acid affinity chromatography (Ni-NTA) using the Amicon^®^ Pro Affinity Concentration Ni-NTA Kit (Merck Millipore, Burlington, MA, USA) rapid centrifugal-based purification method. In the last step, the elution buffer was exchanged for DPBS via ultrafiltration. ^His^dianthin and ^His^dianthin Arg24Ala were additionally purified in a larger batch by combining manual Ni-NTA chromatography and cation exchange chromatography, thus affording pure proteins. Ni-NTA was carried out as described in [62]. Eluted fractions containing the target protein (visualized by SDS-PAGE) were pooled; dialyzed against 50 mM MES Puffer (pH 5.6); and applied to a HiTrap SP XL column connected to an ÄKTA^TM^ start system (GE Healthcare, Chicago, IL, USA). Proteins were eluted with 100 mM, 200 mM, 300 mM and 400 mM of NaCl in 50 mM of MES Puffer (pH 5.6). Buffer exchange and protein concentration of fractions containing the target protein (visualized by SDS-PAGE) were carried out by ultrafiltration (Amicon^®^Ultra 15, 10 K, Merck Millipore, Burlington, MA, USA).

### 5.3. SDS-PAGE and Protein Quantification

Then, 12.5% SDS-polyacrylamide gels were used for SDS-PAGE using the Lämmli method [75]. Protein bands were stained with Coomassie Brillant Blue G250, as described elsewhere [76]. Protein concentrations were determined either by using a bicinchoninic acid assay (Pierce BCA Protein Assay Kit, Thermo Scientific, Rockford, IL, USA) optimized for NanoDrop One (Thermo Scientific, Rockford, IL, USA) or by measuring the absorbance at 280 nm (NanoDrop One, Thermo Scientific, Rockford, IL, USA).

### 5.4. Adenine-Releasing Assay

The *N*-glycosylase activity of different samples was quantified with the adenine-releasing assay. The assay was carried out as described elsewhere [44]. Type I RIPs are able to release adenine from an artificial DNA oligonucleotide 5′-A_30_-3′ (A30) substrate. The assay is based on the quantification of the aforementioned cleaved adenine from the A30 substrate. Therefore, a 169 nM RIP sample was mixed with 21.4 µM of A30 substrate (Metabion International AG, Planegg/Steinkirchen, Germany) and filled up to 50 µL with assay buffer (50 mM of sodium acetate; 100 mM of KCl; pH 5). The mixtures were incubated overnight at 37 °C (for a total of 16.5 h, slightly deviating from the publication). Samples (each 10 µL) were applied to a TLC 0.25 mm pre-coated silica gel 60 glass plate with fluorescent indicator, UV_254_ (Macherey-Nagel, Düren, Germany), and developed by acetonitrile/water/ammonia (32%) (18:1.6:0.6). Released adenine was determined by TLC densitometry at 260 nm using TLC Scanner 4 (CAMAG, Berlin, Germany).

### 5.5. Cell Culture

Neuro-2A cells (DMSZ ACC 148)—mouse neuroblasts with neuronal and amoeboid stem cell morphology isolated from brain tissue—were cultivated in low-glucose (1.0 g/L) Dulbecco’s Modified Eagle Medium (Lonza, Walkersville, MD, USA) supplemented with 10% FBS (Bio&SELL, Feucht/Nürnberg, Germany); 2 mM of alanyl-L-glutamine (UltraGlutamine^TM^ I Supplement, Lonza, Walkersville, MD, USA); and 1% non-essential amino acids (Sigma-Aldrich, St. Louis, USA). HCT116 cells (ATCC^®^ CCL-247^TM^), a human colon cancer cell line, were cultured in McCoy’s 5A Medium (Life Technologies, Carlsbad, CA, USA) supplemented with 10% FBS and 2 mM of glutamine. A2058 cells (ATCC^®^ CRL-11147 ^TM^), a human amelanotic melanoma cell line, were cultivated in high-glucose (4.5 g/L) Dulbecco’s Modified Eagle Medium (Lonza, Walkersville, MD, USA) supplemented with 10% FBS, 2 mM of glutamine and 1% non-essential amino acids. Cells were grown in humidified incubators at 5% CO_2_ and 37 °C.

### 5.6. Cytotoxic Screening of ^His^dianthin Mutants

The cytotoxic screening of ^His^dianthin mutants was monitored by label-free live-cell imaging using the CytoSMART Omni system (CytoSMART Technologies B.V., Eindhoven, the Netherlands). Neuro-2A cells (4000 cells/well) were seeded in 96-well plates containing 100 µL of medium per well. After 24 h, 55 µL of medium was removed and replaced by 55 µL of fresh medium containing 1 nM (final concentration) of ^His^dianthin mutants either supplemented with 1 µg/mL of SO1861 or without supplementation of SO1861 (each in three wells). Control cells were treated with 1 nM of ^His^dianthin ± 1 µg/mL of SO1861 and PBS ± 1 µg/mL of SO1861 (each in three wells). Cells were incubated for a further 48 h. Image analysis was performed using the CytoSMART 33.3 image analysis software package.

### 5.7. Cytotoxicity of ^His^dianthin Arg24Ala in Three Different Cell Lines

The cell viability of ^His^dianthin Arg24Ala was determined in three cell lines: Neuro-2A, HCT116 and A2058 cells. Neuro-2A (4000 cells/well), HCT116 (5000 cells/well) and A2058 (5000 cells/well) were seeded in 96-well plates containing 100 µL of medium per well. After 24 h, 55 µL of medium was replaced by 55 µL of fresh medium supplemented with 0.1 nM and 1 nM of ^His^dianthin Arg24Ala ± 1 µg/mL of SO1861 (final concentrations, each in triplicate). Control cells were treated with 0.1 nM and 1 nM of ^His^dianthin ± 1 µg/mL of SO1861 and PBS ± 1 µg/mL of SO1861 (each in three wells). After adding the samples, cells were incubated for a further 48 h. In addition to the confluence live-cell imaging of the CytoSMART Omni system, the cytotoxicity was evaluated by the MTT endpoint assay [77]. Complete medium was removed from the cells. In total, 100 µL of MTT (Carl Roth, Karlsruhe, Germany) solution (0.5 mg/mL in culture medium) was added to each well. The 96-well plate was incubated at 37 °C for 2 h. To solubilize the formazan product, MTT solution was replaced by 100 µL/well DMSO and further incubated for 30 min. Absorbance was measured at 560 nm (reference wavelength: 620 nm) using the Infinite 200 Microplate Reader (Tecan, Männedorf, Switzerland).

### 5.8. Cytotoxicity of ^His^dianthin Arg24Ala at High Concentrations

Similar to the cytotoxic screening (5.6), Neuro-2A cells (4000 cells/well) were seeded in 96-well plates containing 100 µL of medium per well. After 24 h, the medium was removed and replaced by 55 µL of fresh medium containing ^His^dianthin Arg24Ala ranging from 10 to 1000 nM, either supplemented with 1 µg/mL of SO1861 or without supplementation of SO1861 (final concentrations, each in three wells). Control cells were treated with 10–1000 nM of ^His^dianthin ± 1 µg/mL of SO1861 and PBS ± 1 µg/mL of SO1861 (each in triplicate). Evaluation was carried out as described in Section 5.7.

### 5.9. Fluorescence Labeling

^His^dianthin and ^His^dianthin Arg24Ala (each with a final concentration of 1.5 mg/mL) were labeled with CF^®^568 NHS-ester (10 mM in DMSO; Sigma-Aldrich, St. Louis, MO, USA) according to the manufacturer’s instructions. A dye:protein ratio of 15:1 was chosen. The labeling reaction was purified by ultrafiltration against PBS (Amicon^®^ Ultra-0.5 centrifugal filters, 10 kDa; Merck Millipore, Burlington, VT, USA). Protein concentration and degree of labeling (DOL) of labeled ^His^dianthin and ^His^dianthin Arg24Ala (^His^dianthin-CF568 and ^His^dianthin Arg24Ala-CF568) were determined by measuring absorbances at 280 and 562 nm (Biospectrometer^®^ Basic, Eppendorf, Hamburg, Germany). Fluorescence intensity of labeled proteins was determined at 560/10 nm (excitation) and 595/35 nm (emission) (Infinite 200, Tecan, Männedorf, Switzerland).

### 5.10. Cytotoxicity of ^His^dianthin-CF568 and ^His^dianthin Arg24Ala-CF568

To investigate the impact of the fluorescence label CF^®^568 on ^His^dianthin’s and ^His^dianthin Arg24Ala’s cytotoxicity, 4000 Neuro-2A cells/well were seeded into a 96-well plate containing 100 µL of medium per well. The concentrations used were adapted to the endocytosis assay. After 24 h of incubation, the medium was replaced by 55 µL of fresh medium containing ^His^dianthin and ^His^dianthin Arg24Ala ranging from 0.1 to 100 nM, either supplemented with 1 µg/mL of SO1861 or without supplementation of SO1861 (final concentrations, each in three wells). Control cells were treated with PBS ± 1 µg/mL of SO1861 (each in triplicate). Evaluation was carried out as described in Section 5.7.

### 5.11. Endocytosis Assay

Neuro-2A cells were seeded in 96-well plates and grown for 24 h. To monitor time-dependent endocytosis after 0 h, 2 h, 6 h, 16 h and 24 h, samples were added consecutively in reverse order to the cells. Then, 24 h, 32 h, 42 h, 46 h and 48 h after seeding, corresponding to 24 h, 16 h, 6 h, 2 h and 0 h of incubation with fluorescence-labeled protein, respectively, each 55 µL cell culture medium was removed and replaced by 55 μL of fresh medium containing either ^His^dianthin-CF568 or ^His^dianthin Arg24Ala-CF568 at a final concentration of 100 nM (each in triplicate). Then, 48 h after seeding, the incubation was stopped, and cell culture medium containing non-endocytosed labeled protein was removed. Subsequently, adherent cells were trypsinated (150 μL/well) for 10 min at 37 °C, homogenized and analyzed by flow cytometry (CytoFLEX, Beckman Coulter, Brea, CA, USA). For each sample, at least 10,000 cells were measured, and at least 5000 single cells were included in the analysis. Debris and doublets were excluded from analysis.

### 5.12. Protein Mass Spectrometry

Protein and peptide samples were analyzed by matrix-assisted laser desorption time-of-flight mass spectrometry (MALDI-TOF-MS). Peptides were measured with an Ultraflex II TOF/TOF instrument (Bruker Daltonics, Bremen, Germany) equipped with a 200 Hz solid-state Smart beamTM laser, while the labeled protein was measured on a Bruker UltrafleXtreme instrument. Data were analyzed using the FlexAnalysis 2.4 software provided with the instruments. Samples were applied via the dried-droplet technique. Peptides were generated by trypsin or AspN in-gel digestion following a protocol described elsewhere [78]. The mass fingerprints of the generated peptides were recorded in positive reflector mode (RP_PepMix) over a *m*/*z* range of 600–4000. α-cyano-4-hydroxycinnamic acid was used as matrix. Intact labeled proteins were measured on sinapinic acid as matrix in positive linear mode over a *m*/*z* range of 5000–55,000.

For native MS measurements, ^His^dianthin was buffer-exchanged by dialysis into 50 mM of ammonium acetate buffer, pH 6.24. A stock solution of SO1861 (537 µM, in water) was first diluted to 1:2 with 100 mM of ammonium acetate to reach 50 mM of ammonium acetate and was then adjusted with 50 mM of ammonium acetate to a final concentration of 200 µM of SO1861. The diluted stock solution was mixed with ^His^dianthin to obtain final concentrations of 10 µM of ^His^dianthin and 30 µM, 50 µM or 100 µM of SO1861. For the measurement under denaturing conditions, a 5 µM dilution of ^His^dianthin in 0.1% formic acid in acetonitrile:water (1:1) was used. The native MS measurements were performed on a Q Exactive HF mass spectrometer using direct injection with an offline nanoESI source head on the NanoFlex ion source (Thermo Scientific). The intact protein mode was activated, and the trapping gas pressure was set to 0.2 (available with Biopharma option). All spectra were recorded for at least 30 s in the profile mode with positive polarity using the following settings: scan range, 300 to 4000 *m*/*z* (500 to 2000 *m*/*z* for denaturing conditions); resolution, 15,000; microscans, 5; AGC target, 3e6; maximum inject time, 200 ms; spray voltage, 2.2 kV; capillary temperature, 175 °C; S-lens RF level, 60; in-source decay (ISD), 100 eV. The software tool UniDec 6.0.3 was used for data processing and spectral deconvolution using the default settings with the following modifications: charge range, 5–20 (27–35 for denaturing conditions); mass range, 29–32 kDa; sample mass, every 1 Da [79]. 

### 5.13. Statistical Analysis

All experiments were conducted in duplicate or in triplicate and were repeated three times independently. The obtained data were analyzed using RStudio (RStudio Team (2020); RStudio: Integrated Development Environment for R. RStudio, PBC, Boston, MA, USA) All data were first tested for normal distribution using the Shapiro–Wilk test. Outliers of normally distributed data were identified using Grubbs’ test and excluded from the analysis. Statistical significance of normally distributed data was determined using Student’s *t*-test. Non-normally distributed data were tested for significance using the Wilcoxon–Mann–Whitney *U* test. All values are presented as arithmetic means ± standard deviation.

## Figures and Tables

**Table 1:** Primers used for site-directed mutagenesis of ^His^dianthin. Underlined bases represent the mutated nucleotides that cause the exchange of arginine to alanine or lysine to alanine at the protein level. Primer sequences and their annealing temperatures (Ta) were generated with NEBaseChanger^®^ (New England Biolabs, Ipswich, MA, USA). Primers were synthesized by Metabion (Planegg/Steinkirchen, Germany).

Mutation	Sequence	Ta (°C)
Arg24Ala	forward 5′-GGA TCA AAT CGC AAA CAA TGT GAG G-3′	56
	reverse 5′-AGA AAA GAT GAG TAT TGA CTC-3′	
Lys125Ala	forward 5′-CTT GGT TAC CGC GAA CTT CCC AAA C-3′	58
	reverse 5′-TTC TGT ATG TAC CTA AAT CG-3′	
Lys227Ala	forward 5′-CGT GTT TAA TGC AGA TTA TGA TTT CGG G-3′	59
	reverse 5′-AGA AAA GAT GAG TAT TGA CTC-3′	

## Data Availability

The raw data supporting the conclusions of this article will be made available by the authors on request.

## Supplementary Materials

The following supporting information can be downloaded at https://www.mdpi.com/article/10.3390/toxins16050219/s1: Figure S1. PCR products of side-directed mutagenesis of pET11d-^His^dianthin; Figure S2. MALDI mass spectra allowing the identification of ^His^dianthin Arg24Ala; Figure S3. ESI-MS spectrum of ^His^dianthin measured under denaturing conditions (0.1 % formic acid in acetonitrile/water (1:1)); Figure S4. MALDI mass spectra of the labeling reaction of ^His^dianthin and ^His^dianthin Arg24Ala with the fluorescent dye CF®568; Figure S5. Cytotoxicity of ^His^dianthin-CF568 and ^His^dianthin Arg24Ala-CF568.
