# Supplementary material for: Mutational Analysis of RIP Type I Dianthin-30 Suggests a Role for Arg24 in Endocytosis
Source: Toxins (Basel). 2024 May 10;16(5):219. doi: 10.3390/toxins16050219 (PMC11125672; doi:10.3390/toxins16050219)
Supplement: Supplementary file 1 [file toxins-16-00219-s001.zip › toxins-2868251-supplementary.pdf]

Mutational analysis of type I RIP dianthin-30 reveals function of Arg24 in endocytosis

Louisa Schlaak, Christoph Weise, Benno Kuropka, Alexander Weng

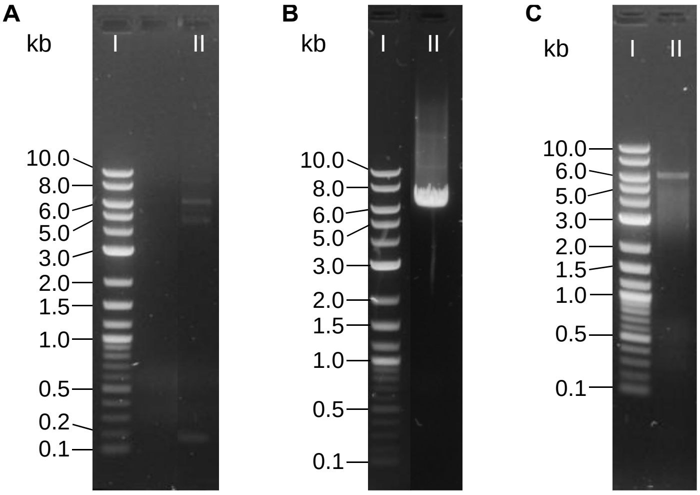

Figure S1. PCR products of side-directed mutagenesis of pET11d-^{His}dianthin. For the 1 % agarose gels, the 1 kb plus DNA ladder (New England Labs, Ipswich, USA) was used as marker (lane I). All three target vectors showed a band in the range of the expected size of 6114 bp. (**A**) Lane II: pET11d-^{His}dianthin Lys195Ala (**B**) Lane II: pET11d-^{His}dianthin Lys227Ala (**C**) Lane II: pET11d-^{His}dianthin Arg24Ala.

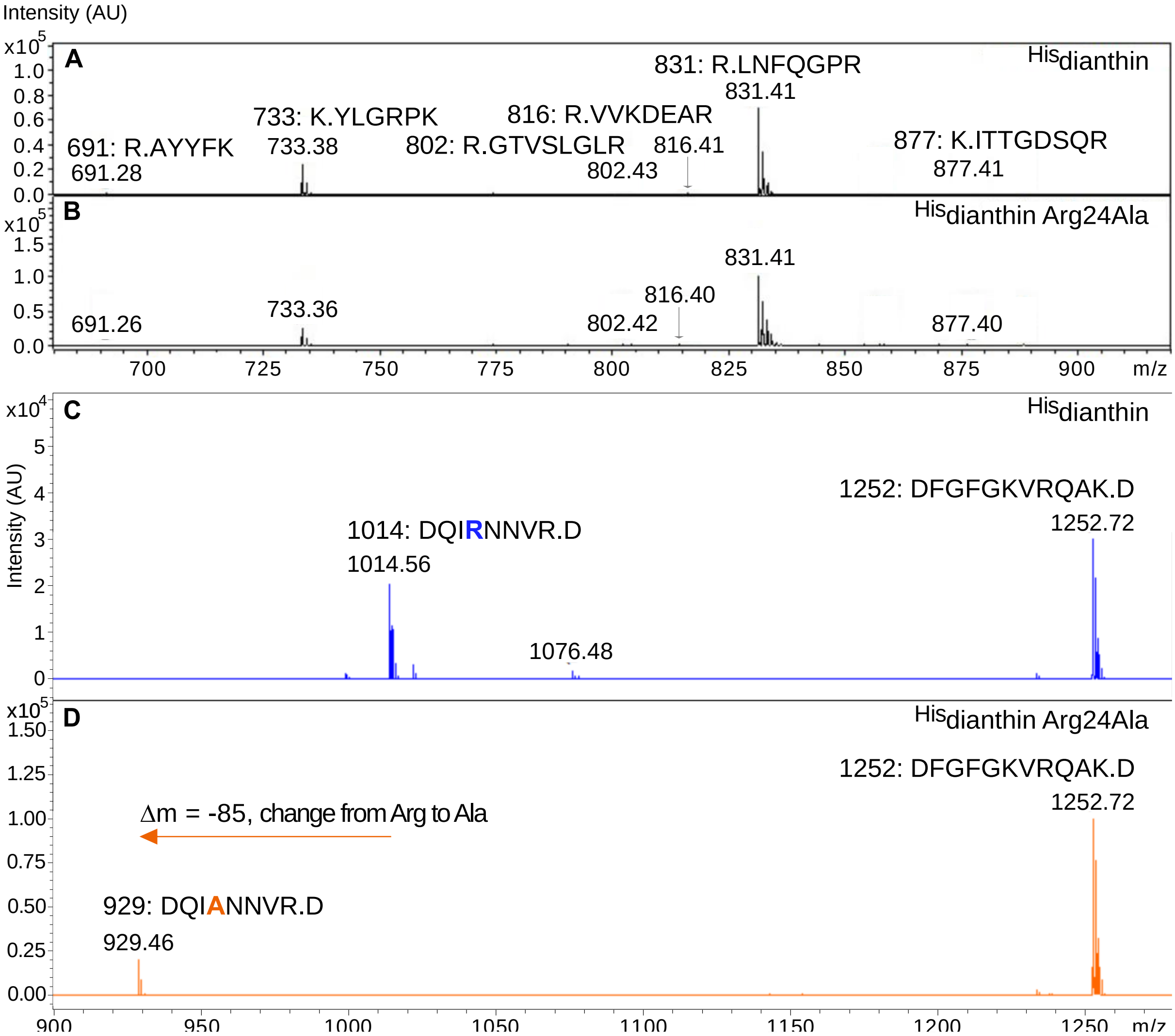

Figure S2. MALDI mass spectra allowing the identification of ^{His}dianthin Arg24Ala. (**A**) & (**B**) Tryptic peptide mass fingerprints of ^{His}dianthin and ^{His}dianthin Arg24Ala in the mass range of 680–920 m/z. All expected peptides of ^{His}dianthin and ^{His}dianthin Arg24Ala matched. Thus, the identity of ^{His}dianthin Arg24Ala as a ^{His}dianthin variant was confirmed. (**C**) & (**D**) Peptide mass fingerprints of in-gel digestion with AspN in the mass range of 900–1280 m/z. AspN cleaves *N*-terminally on aspartic acid. While the resulting peptide covering Arg24 of ^{His}dianthin was detected at 1014.56 m/z, the peak shifted by -85 Da to 929.46 m/z for ^{His}dianthin Arg24Ala. 85 Da corresponded to the mass difference expected from alanine substitution.

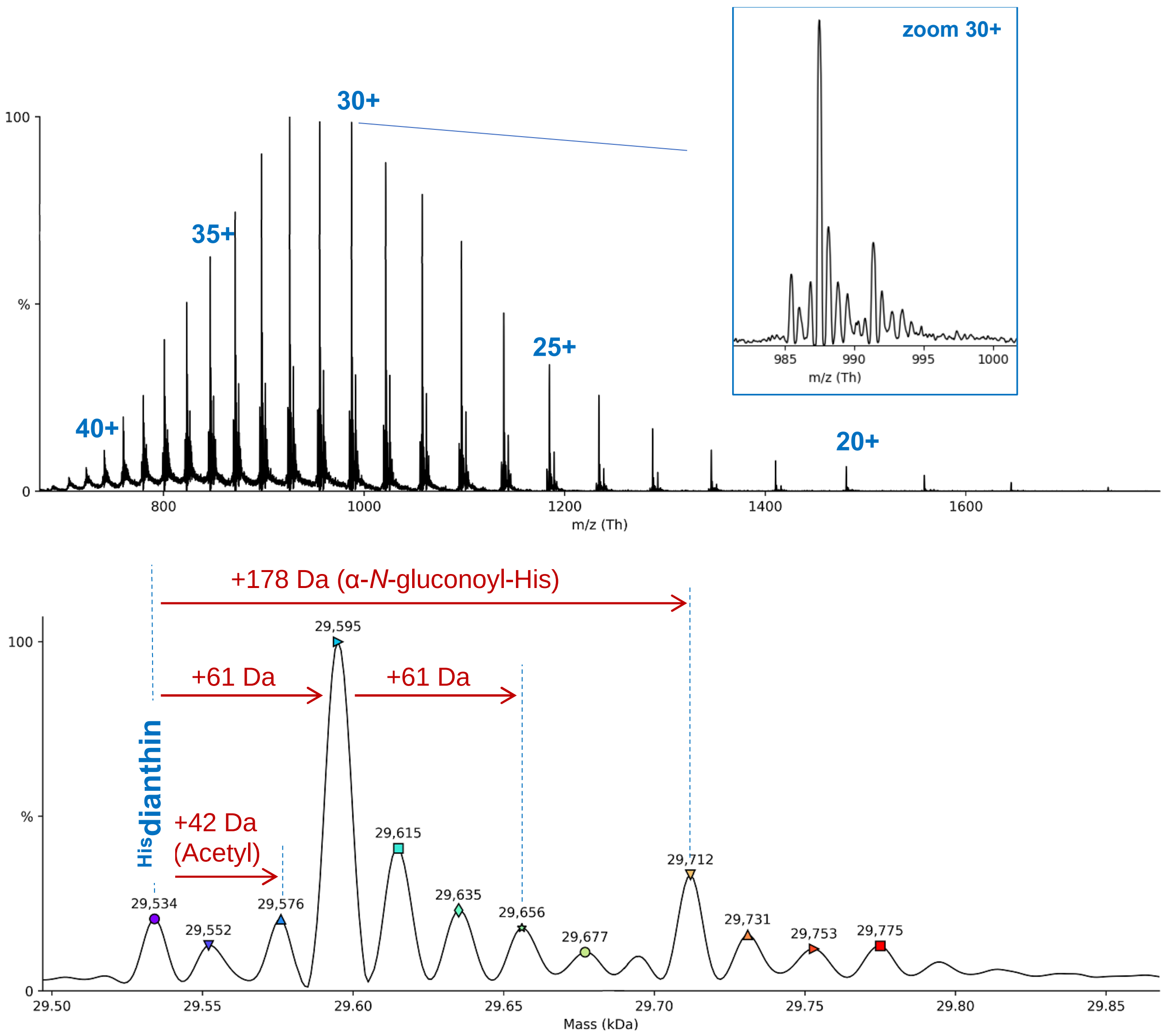

Figure S3. ESI-MS spectrum of ^{His}dianthin measured under denaturing conditions (0.1 % formic acid in acetonitrile/water (1:1)). The raw spectrum (top) shows a typical wide charge state distribution of an unfolded protein. The inset shows a zoom of charge state 30+, indicating some heterogeneity. Spectral deconvolution using the software Unidec shows the experimentally determined masses (bottom). The signal measured at 29,534 Da matches the theoretical mass of ^{His}dianthin if the *N*-terminal methionine is removed (calculated MW: 29,533.3 Da). The signal at 29,576 likely corresponds to an *N*-terminal acetylation. The signal at 29,712 likely corresponds to *N*-gluconoylation of the His-tag, which is commonly observed for His-tagged proteins expressed in *E. coli*. The dominant peak at 29,595 is ~61 Da higher compared to the calculated mass of ^{His}dianthin and likely corresponds to an unknown modification, with a second peak at 29,656 likely corresponding to the doubly modified protein.

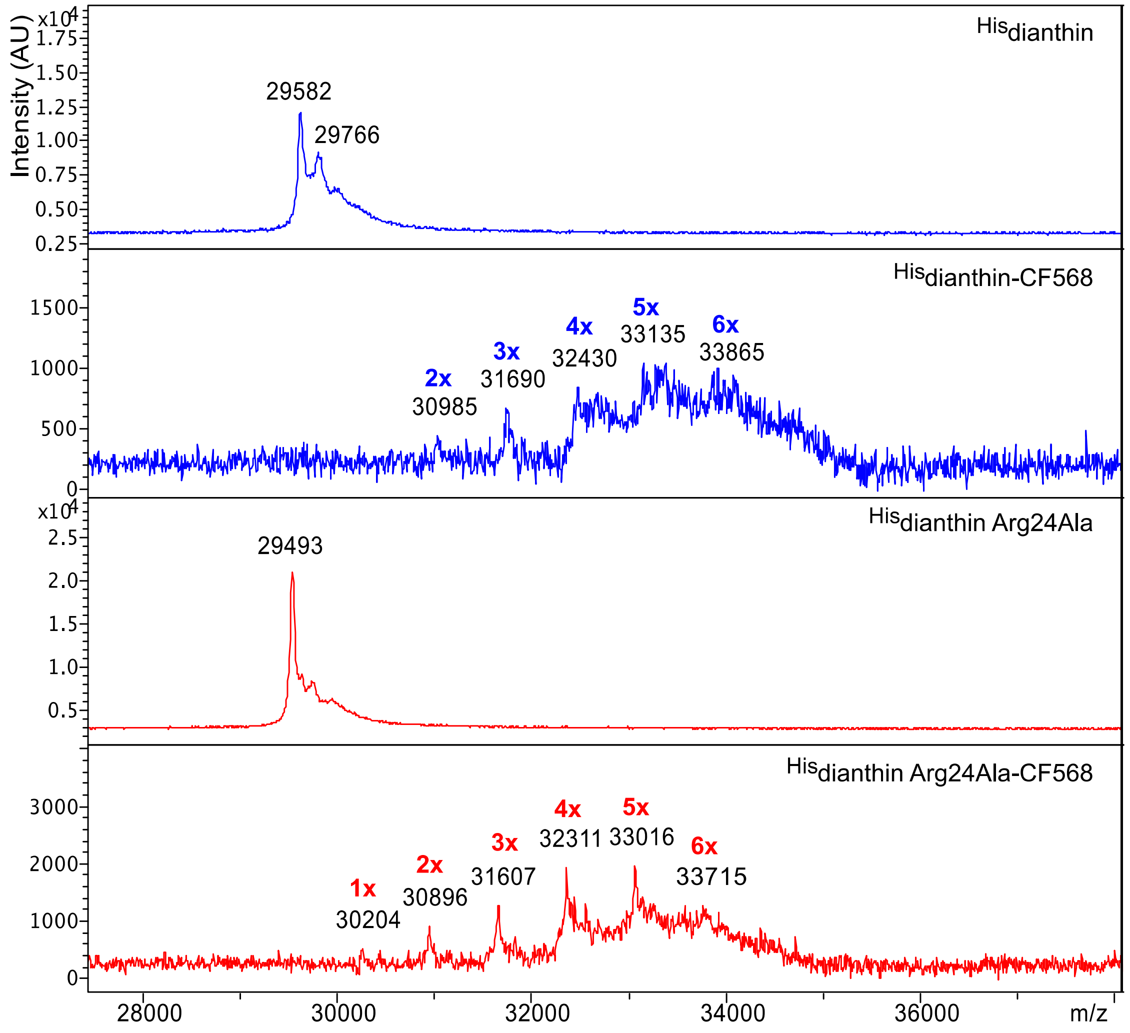

Figure S4. MALDI mass spectra of the labeling reaction of ^{His}dianthin and ^{His}dianthin Arg24Ala with the fluorescent dye CF®568. MS spectra were recorded from both the unlabeled proteins and the labeled products. ^{His}dianthin (29582 Da; 29766 Da corresponds to *N*-gluconoylated ^{His}dianthin) and ^{His}dianthin Arg24Ala (29493 Da) were fully converted to the labeled product (absence of the corresponding mass peaks in the mass spectra of the products). CF®568 reacted with ^{His}dianthin and ^{His}dianthin Arg24Ala in several stoichiometric ratios. Mass of ^{His}dianthin-CF568 ranged from 30985 Da to 33865 Da which corresponded to the addition of 2–6 color molecules per protein molecule. Mass of ^{His}dianthin Arg24Ala-CF568 ranged from 30204 Da to 33715 Da, corresponding an addition of 1–6 color molecules per protein molecule. Calculated over the first five adducts, there was an average mass increase of 698 Da, which corresponded to the mass of the fluorescent dye after cleavage of oxygen.

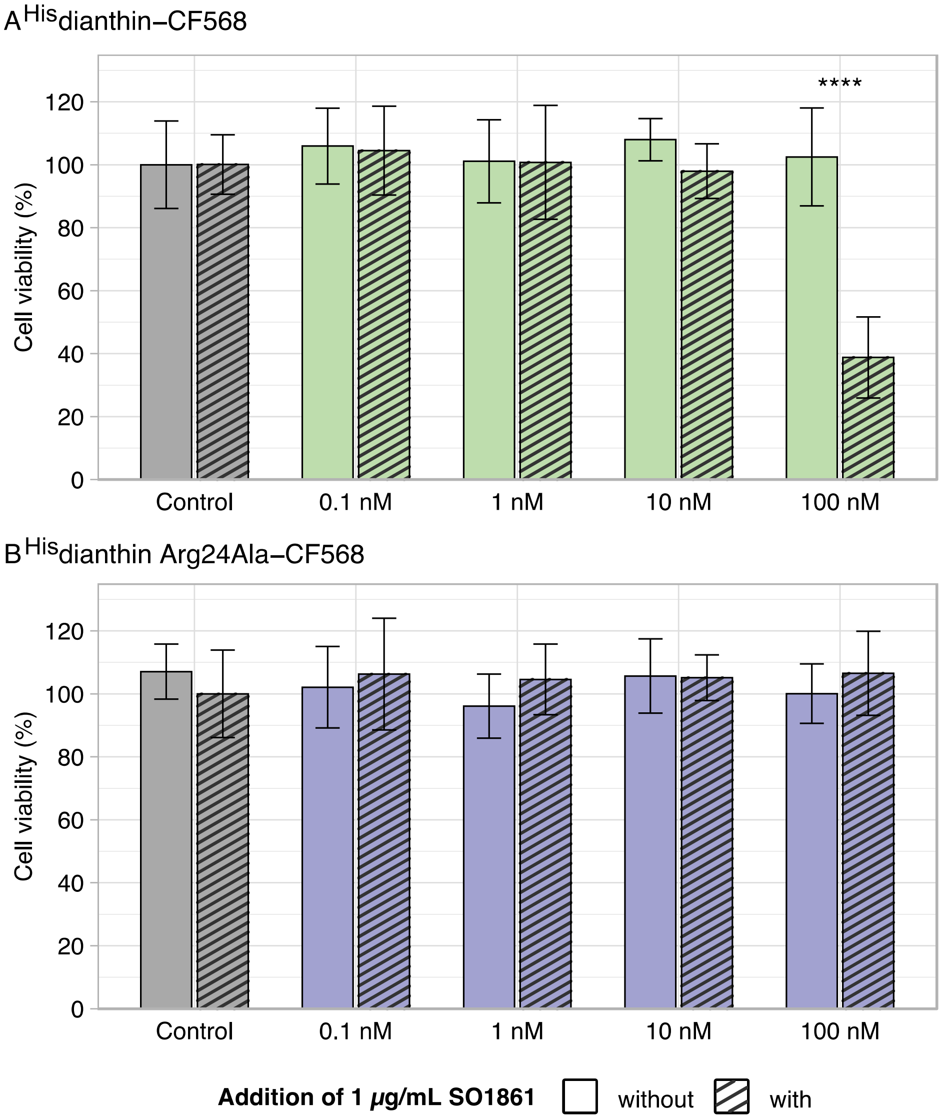

Figure S5. Cytotoxicity of Hisdianthin-CF568 and Hisdianthin Arg24Ala-CF568. Neuro-2A cells were incubated with 0.1 nM, 1 nM, 10 nM and 100 nM Hisdianthin-CF568 and Hisdianthin Arg24Ala-CF568 ± 1 μg/mL SO1861 for 48 h. Control cells were treated equivalently with PBS ± 1 μg/mL SO1861. (**A**) Hisdianthin-CF568 + SO1861 was significantally cytotoxic only at 100 nM. (**B**) The labelling with fluorescent dye caused for Hisdianthin Arg24Ala-CF568 a complete los of cytotoxicity in the concentration range between 0.1 to 100 nM. Shown are the means ± standard deviation of three independent measurements, n = 3 (Significance: ****, $p \leq 0.0001$; Student's *t*-test).
